# Pancreatic cancer tumor microenvironment is a major therapeutic barrier and target

**DOI:** 10.3389/fimmu.2024.1287459

**Published:** 2024-02-01

**Authors:** Conner Hartupee, Bolni Marius Nagalo, Chiswili Y. Chabu, Mulu Z. Tesfay, Joycelynn Coleman-Barnett, John T. West, Omeed Moaven

**Affiliations:** ^1^ Division of Surgical Oncology, Department of Surgery, Louisiana State University (LSU) Health, New Orleans, LA, United States; ^2^ Department of Pathology, University of Arkansas for Medical Sciences (UAMS), Little Rock, AR, United States; ^3^ The Winthrop P. Rockefeller Cancer Institute, University of Arkansas for Medical Sciences (UAMS), Little Rock, AR, United States; ^4^ Division of Biological Sciences, University of Missouri, Columbia, MO, United States; ^5^ Department of Surgery, School of Medicine, University of Missouri, Columbia, MO, United States; ^6^ Siteman Cancer Center, Washington University, St. Louis, MO, United States; ^7^ Department of Interdisciplinary Oncology, Louisiana Cancer Research Center, Louisiana State University (LSU) Health, New Orleans, LA, United States; ^8^ Louisiana State University - Louisiana Children's Medical Center (LSU - LCMC) Cancer Center, New Orleans, LA, United States

**Keywords:** pancreatic adenocarcinoma, tumor microenvironment, PDAC TME, tumor immune microenvironment, immune therapeutics, cancer immunology

## Abstract

Pancreatic Ductal Adenocarcinoma (PDAC) is projected to become the 2nd leading cause of cancer-related deaths in the United States. Limitations in early detection and treatment barriers contribute to the lack of substantial success in the treatment of this challenging-to-treat malignancy. Desmoplasia is the hallmark of PDAC microenvironment that creates a physical and immunologic barrier. Stromal support cells and immunomodulatory cells face aberrant signaling by pancreatic cancer cells that shifts the complex balance of proper repair mechanisms into a state of dysregulation. The product of this dysregulation is the desmoplastic environment that encases the malignant cells leading to a dense, hypoxic environment that promotes further tumorigenesis, provides innate systemic resistance, and suppresses anti-tumor immune invasion. This desmoplastic environment combined with the immunoregulatory events that allow it to persist serve as the primary focus of this review. The physical barrier and immune counterbalance in the tumor microenvironment (TME) make PDAC an immunologically cold tumor. To convert PDAC into an immunologically hot tumor, tumor microenvironment could be considered alongside the tumor cells. We discuss the complex network of microenvironment molecular and cellular composition and explore how they can be targeted to overcome immuno-therapeutic challenges.

## Introduction

Pancreatic Ductal Adenocarcinoma (PDAC) is currently the 4^th^ leading cause of cancer-related deaths in the United States (1). It is projected that by 2030 it will become the 2^nd^ leading cause of cancer-related deaths (2). Although advancements are made in the treatment of resectable disease, this population makes up only 15-20% of new pancreatic cancer cases as most patients do not present until late into the disease course, at which point it is not resectable. Due to the nature of this disease, systemic treatment is an essential component of the multimodality treatment of pancreatic cancer (3). Unfortunately, primary and secondary resistance are very common in PDAC and there is an urgent need for developing therapeutic modalities with adequate oncologic benefit and durability. Immunotherapy has recently revolutionized the treatment of various cancers but repeat trials have shown PDAC is relatively unresponsive to such therapies.

There is a growing interest in focusing on the tumor microenvironment (TME) and its role in resistance to systemic therapies and as a target for more effective therapeutic modalities. Pancreatic adenocarcinoma, along with other solid tumors, is what is best described as an “immunologically cold” tumor. PDAC lacks excessive mutations that would allow the body’s immune system to recognize the tumor cells due to tumor specific antigens (4). Due to the limited immunogenicity of PDAC, this leads to insufficient cancer antigen presentation, resulting in a notably weak or even absent immune response and inadequate T-cell trafficking (5). Additionally, PDAC reprograms surrounding tissue and immune cells to create a dense, fibroblastic environment with limited anti-tumor immunity. Previous studies have evaluated the function and composition of the tumor microenvironment (TME) to better understand contributing factors to the therapeutic challenge of PDAC and to answer the question of why PDAC has poor immunogenicity. The PDAC TME includes a mixture of cancerous pancreatic cells, support cells, immune cells, and dense extracellular matrix. Even within this complex microenvironment, we see variability between one tumor’s molecular and genetic profile vs. another that results in differing clinical outcomes (6, 7). This heterogeneous system works in conjunction to provide an environment that remains relatively impermeable to treatment, adaptive to therapies, and supportive of cancerous spread.

## Pancreatic cancer cells

Pancreatic cancer cells (PCC) arise from either ductal pancreas cells or acinar pancreas cells. Acinar cells perform the role of digestive enzyme production and ductal cells secrete bicarbonate ions and water needed to alkalize and activate the enzymatic secretions of the pancreas. Some debate remains as to the exact cell-of-origin but recent literature indicates the origin may vary based on the number and type of initial mutations seen with the tumor environment (8). The significance of the contextual difference in the cell-of-origin may eventually point us towards a better understanding of the biological function of these tumors and patient outcomes as seen with similar solid tumor cancers (9).

Formation of pancreatic cancer cells is believed to involve processes related to chronic inflammation, hypoxia, and the accumulation of repetitive mutations. These factors collectively contribute to the development of pancreatic intraepithelial neoplasia (PanIN). In the process of inflammation, an initial, acute inflammatory reaction drives the migration of neutrophils, natural killer cells, dendritic cells, and macrophages to the site of insult (10). The innate immune system in attempting to resolve the initial acute insult promotes a cascade of signaling that recruits the adaptive immune response along with stimulation of endogenous repair mechanisms to reestablish the structure and function of the surrounding tissue. When the acute inflammatory response begins to maladaptively produce a chronic inflammatory response, the combination of reactive oxygen species, continued tissue injury, and immunosuppressive functions of the chronic inflammation leads to an environment prone to oncogenic mutations (10).

Over time these pathologic insults will alter the genetic signature of the surround tissue and this neoplastic tissue accumulates a significant number of mutations that transforms lesions from a precancerous stage into cells more consistent with PDAC. *Kirsten Rat Sarcoma* (*KRAS*) presents as a consistent oncogenic mutation with inactivation of the GTPase activity of the KRAS protein complex leading to consistent activation and downstream signaling of key proliferative pathways like mTOR, NF-kB, and ERK (11). Further evidence supports that *KRAS* mutations are a key instigator in upregulation of glycolytic metabolic reprogramming and Sonic Hedge Hog (SHH) mediated activation of pancreatic stellate cells (12, 13).

*Oncogenic KRAS* mutation alone does not guarantee neoplastic formation, as cell populations positive for *KRAS* were observed to be non-proliferative within normal pancreatic tissue. These cells were instead signaled to cease proliferation through a mechanism known as oncogene-induced senescence. The physiologic role of this senescence is innately to protect against oncogenesis within these cells. This senescent state may be bypassed by proinflammatory mechanisms, stemming from pathologic events such as pancreatitis, that reactivate the proliferation of pre-neoplastic cells, enabling further mutagenesis (14). Senescent cell populations cease proliferation but remain as active components of the tissue microenvironment. Many of these senescent cells fall under what is termed senescence-associated secretory phenotype (SASP) and release a variety of pro-inflammatory molecules including growth factors, interleukins, cytokines, and proteases(15, 16). The intent of these signaling molecules is designed to awaken the immune system to eliminate said senescent cells; however, senescent cells release facts that act autonomously to accelerate cell proliferation and epithelial to mesenchymal transition (EMT). Additional mechanisms of cellular damage, including chemotherapeutics and radiotherapy, have been shown to induce a tumor-supportive senescence-like secretory phenotype in the TMEs, demonstrating therapy-resistant malignancies as these populations do not undergo apoptosis (17). A relatively new role within the neoplastic environment, senescent cells prior to oncogenesis and throughout a malignancy has been identified as a significant component of the TME (18).

Additional mutations, such as deactivating mutations in p53 lead to uninhibited collection of DNA damage and SMAD4 inactivation, leading to unregulated TGF-β signaling. Upregulation of TGF-β, a molecule present throughout the PDAC TME initially contributes to tumor suppression through apoptosis and cell-cycle arrest (19). Although the inactivation of SMAD4 and progression of PanIN leads to TGF-β functioning as a tumorigenic marker that promotes progression and EMT. These mutations function as the beginning of a neoplastic group of genes necessary but not always present within PDAC genetics. Additional precursor mutations of *Her-2*, *p16*, and *BRCA2* have been detected within the PanINs as grading successively increases (20). Proto-oncogenic and oncogenic mutations combined with a reduction in response by the immune system to properly suppress precancerous cells leads to an environment of where normal regulatory mechanisms are compromised (21). Such precancerous cells inevitably alter the surrounding environment to isolate and support their growth concomitant with immune suppression to minimize interference. Part of this transition is directed by the signaling pathways evidenced in PCC mutations and signaling.

Noted to release a variety of chemokines and cytokines, PCCs play an essential role in the recruitment of support cells and manipulation of immune cells. PCCs directly signal pancreatic stellate cells (PSCs) for activation by means of key molecules such as transforming growth factor-beta (TGFβ1), platelet-derived growth factor (PDGF), and fibroblast growth factor-2 (FGF-2) leading to increased fibrogenesis by the PSCs (22). PCCs are shown to produce excess chemokines that are heavily involved in the activation of Cancer-associated fibroblasts, including CXCL1, -5, and -8 binding to fibroblasts’ CXCR2 receptors, leading to increased fibroblast activation and reprogramming (23). IL-1β, a traditionally pro-inflammatory cytokine, is shown to be released by PCCs *in vivo* early within tumorigenesis and is linked to recruitment and activation of both PSCs and immune suppressive cell types (24). The chemokines CSF-1 (also known as M-CSF), CSF-2 (also known as GM-CSF), and CSF-3 (also known as G-CSF) are also shown to be expressed by PCCs, leading to decreased concentrations of anti-tumor dendritic cells and increased concentrations of immune suppressive cells within the TME (25, 26). In large part, oncogenic mutations play a key role in upregulating the expression of proteins that allow PCCs to tailor the environment to reduce anti-tumor cell populations. Pre-cancerous cells that fail to express these proteins are potentially reduced by proper immune responses prior to becoming PanINs, hence, we see consistently high levels of *KRAS* and *p53* mutations in PCC genetics.

Further, PCCs suppress immune components in PDAC TME via the release of signaling exosomes. PCCs release exosomes containing miRNA155 and miRNA1290, into cancer-associated fibroblasts(CAFs), leading to elevated fibrogenesis and resulting into highly fibrotic TME (27, 28). The fibrotic milieu generate a tissue mechanical landscape that promotes adaptation of PDAC cancer cells, contributing to robust tumorigenesis.

Low O_2_ saturation in the PDAC TME results in overexpression of hypoxia-inducible factors (HIFs). These proteins act as homeostatic transcription factors in a cascading response to counteract and adapt to the negative environmental stimuli the cells are in, producing VEGF, altering metabolic pathways to prioritize glycolysis, and upregulation of chemoresistant proteins like MDR1 and HGF (29, 30). Additionally, HIF1α/2α induces miRNA301a which leads to M2 macrophage polarization resulting in pro-tumorigenic downstream processes (31). NADPH Oxidase (NOX), a transmembrane protein with roles in multiple signaling pathways, is shown to be overexpressed in cancer cells and associated with poor clinical outcomes due to its role in ROS production and utilization in low oxygen environments (32). The cancer cells readily adapt to the hypoxic environment they create, and the additional metabolic changes further promote EMT and metastasis.

EMT, a key feature of malignant progression, is the loss of epithelial features and dedifferentiation to a mesenchymal phenotype. The mesenchymal phenotype is consistent with embryonic stem cells and supports migration and rapid proliferation. Down-regulation of the cell-to-cell adhesion marker, e-cadherin, along with upregulation of n-cadherin and vimentin denote EMT progression (33). Key transcription factors traditionally upregulated in PDAC mesenchymal cells includes SNAIL, SLUG, TWIST1, Zeb1, and 2 (34). Many of such transcription factors being linked to increased mobility and commonly observed in circulating tumor cells. Throughout the formation of the TME and malignant progression of the disease a series of complex interactions occur between the surrounding TME and PCCs much of which is discussed within this review. By understanding these cellular changes, we gain insights into how pancreatic cancer progresses. This knowledge and the current progress of research will continue to guide the development of strategies to halt or slow down the disease.

## Pancreatic stellate cells

Pancreatic stellate cells function as a key component within all aspects of the pancreatic tumor microenvironment. Discovered in 1998 within rat pancreas, these cells work to maintain the proper tissue environment within the pancreas through insults and injury(35). Quiescent PSCs are characterized by high vitamin A, desmin, vimentin, and GFAP levels with little migration or proliferation (36). These cells maintain the extracellular matrix through protein production and basement membrane stabilization, and in times of physiologic stressors, may play a role in autoimmune regulation (37–39) Known triggers of PSCs include ethanol, high glucose, inflammation, reactive oxygen species, and the inflammatory markers produced in local pathologies (40). Transforming Growth Factor-Beta (TGF-β1), CTGF, PDGF, IL-1, TNF-A, AP-1, activin-a, and HIF-1 have been established as direct markers of activation, while PPAR-g, IL-10, SMAD7, and retinoic acid are markers of inhibition (40). PDAC is characterized by increased disproportionate expression of many activating markers through chronic inflammation and hypoxia resulting in the chronic and malignant activation of PSCs.

These activated PSCs function as the architects of extracellular matrix deposition and pancreatic tissue remodeling that is closer in nature to what is seen in pancreatic cancer. Activated PSCs are the primary contributor to the dense, fibrotic environment that supports the growth of pancreatic cancer cells. Transforming into a myofibroblast-like characteristic, PSCs produce and release a variety of ECM remodeling proteins including collagens, periostin, fibronectin, Matrix Metalloproteinases (MMPs) and a decreased release of Tissue Inhibitors of Metalloproteinases (TIMPS) (41). In healthy pancreas stroma, moderate production of these ECM proteins is balanced with appropriate breakdown and recycling of ECM tissue to allow moderation and turnover. The ratio of proteins deposited by PSCs becomes a determining factor in PDAC phenotypes translating to increased tumor progression (42). The disproportionate deposition of these fibrotic proteins in the PDAC TME promotes hydraulic compression 2-3x the normal pressure of the surrounding environment with a poorly perfused vascular supply (43, 44). The increased pressure and decreased vascular flow also lends itself to an environment less susceptible to local delivery of chemotherapeutic agents and antibody penetration (45, 46). The isolated environment developed by dysregulated protein deposition results in an unnatural encasement that isolates and protects the TME known as the desmoplastic reaction.

The role of PSCs in immune response modulation within the context of PDAC is notable alongside the fibrotic environment they create. PSCs exhibit a complicated influence on the immune landscape of the tumor microenvironment. Releasing Interleukin-6 (IL-6), which orchestrates the activation and migration of Myeloid-Derived Suppressor Cells (MDSCs) into the TME with additional consequent immunosuppression discussed further in the text (47). Additionally, activated PSCs contribute to immune regulation by releasing CXCL12, a factor responsible for sequestering CD8 T-cells, thereby hampering their inherent antitumor activity. Another aspect of PSC-mediated immune modulation involves the production of Galectin-1, a protein associated with diverse effects, including the induction of CD3+ T-cell apoptosis, release of TH2 cytokines, and evasion of immune cell responses (48, 49). Further research has underscored PSCs’ involvement in extracellular vesicle signaling, revealing the upregulation of CXCL10 in PSCs during co-culture with pancreatic cancer cells (PCCs), which suggests a crosstalk mechanism contributing to the recruitment of FoxP3+ Tregs (50). PSCs have a potentially significant influence over the initiation and maintenance of immune responses in PDAC.

The immunosuppressed shelter of the desmoplastic environment works in tandem with the role of PSCs in signaling support of PCCs. A combination of signaling molecules have been linked to increased growth of PDAC tumors along with further metastasis and chemoresistance that allows the PDAC TME to continue unchecked. A range of growth factors released by PSCs include TGF-β, Platelet-Derived Growth Factor (PDGF), and Vascular Endothelial Growth Factor (VEGF) (51). Along with growth factors, signaling pathways including WNT/B-Catenin are upregulated in PDAC cancer cells by PSC-bound glutamine synthetase when co-cultured (H. 52). Similarly, PDAC-triggered autophagy by GPT1 in PSCs provides an alanine fuel source to support metabolic pathways in PCCs, allowing growth and support for further spread (53).

Additionally, PSCs exert their impact on PCCs’ therapeutic responses, where the production of Hes1 by PSCs emerges as a determinant of chemoresistance via overactivation of the NOTCH signaling pathway, as elucidated by recent findings (54). Furthermore, the extracellular matrix (ECM) protein Periostin emerges as a key regulator, intricately linked to PDAC progression. Periostin, an ECM protein functioning in tissue remodeling, is found to be linked to increased proliferation and metastasis in PDAC, and when suppressed in spheroid models exhibited increased PDAC toxicity by Natural Killer Cells (55). Metastasis and further tumor progression are also benefited from PSCs due to other factors below.

PSCs play a key role in further carcinogenesis and evolution of PDAC. PSC-derived fibrosis and signaling pathways overexpressed by PSCs influence the EMT process as seen by previous research. Secretion of IL-6 and utilization of the STAT3/NRF2 pathway influences the PDAC EMT (56). Similar effects are seen with CD10+ PSCs explicit MMP3 release as a method of surrounding tissue breakdown to provide local tissue invasion (57). Kindlin-2 expression is also linked to PDAC progression through downregulating HOXB9 and E-cadherin, leading to increased metastasis (58). MiRNA-21 exosomal release by PSCs linked to enhancing Ras/ERK pathways and PDAC cancer cell migration (59).

Focused therapies targeting the role of PSCs in the PDAC TME are gaining traction as the library of evidence indicating their pro-tumorigenic role grows. Targeted inhibition of the pathways and molecules discussed previously have been referenced in the current literature, with some results more promising than others. Some limitations include the expandability from *in vitro*/*in vivo* models to clinical trials, which has shown limited success. One such article notes the promise of early intervention in the PanIN pathway with targeted inhibition may function as a curative approach; however, given the clinical presentation of late-stage disease makes such an approach currently infeasible.

Studies have shed light in therapeutic potential in repolarization of activated PSCs into a quiescent state. Vitamin A analogs have resulted in inactivation of PSCs in chronic pancreatitis that may protect the pancreatic tissue microenvironment (60). Calcipotriol, a vitamin D analog, has also been shown to reduce TGF-β and SMAD4 signaling with increased Meflin expression, assisted in stromal reprogramming with increased gemcitabine efficacy (61, 62). Similarly, BET inhibitors (JQ1/I-BET151) has been shown to induce PSC quiescence and reduce production of collagen 1 and PDAC fibrosis (63). The key to treatment may be in attenuation of overall PSC function rather than complete removal or targeted inhibition. Exploring which aspects of the ECM that PSCs produce and limiting the effects of over-expressed pathways may present a solution, but accessing and selectively targeting these pathways in clinical models remains a challenge.

## Cancer-associated fibroblasts

In conjunction with PSCs, Cancer-Associated Fibroblasts (CAFs) are recognized as a primary contributor to the PDAC TME through generation of many of the extracellular matrix proteins seen. PSC differentiation into CAFs represents a portion of the suspected cells, approximately 15%, of origin involved in PDAC, with other proposed cells including endothelial cells, mesothelial cells, macrophages, stem cells, and a growing list of other cells (64–67). The significance of this literature suggests that malignant reprogramming through PCC signaling induces fibroblast-like phenotypes out of various cell lines depending on the location and context of PDAC.

Common markers for identifying CAFs include Collagen 1 Type A1 (COL1A1), Fibroblast activation Protein (FAP), Podoplanin (PDPN), and Decorin (DCN) (68, 69). Current literature categorizes CAFs into three main subsets, each with distinct markers and functions. A subset of CAFs that potentially presents a direct link to PSCs is Myofibroblastic CAFs (myCAFs), stimulated by TGF-β signaling, which cluster around PDAC cancer cells and are characterized by their expression of alpha-smooth muscle actin (a-SMA) and TGF-β signaling. These myCAFs are believed to contribute significantly to the dense extracellular matrix (ECM) observed in the TME. Inflammatory CAFs (iCAFs), on the other hand, are influenced by IL-1 signaling and are located at the periphery of the TME. These iCAFs exhibit signaling involving IL-6, IL-8, and CXCL1/2/9/12 and are thought to play a role in immunosuppression and tumorigenesis (70, 71). The component balance between the ratio of myCAFs and iCAFs has also been shown to indicate tumorigenesis. Increased TGF-β and SHH signaling increases the myCAF to iCAF ratio predicts slower tumor growth, while a higher iCAF to myCAF ratio leads to the opposite (72, 73).

Adding to this complexity, a subset known as complement-secreting CAFs (csCAFs), unique to the PDAC TME, exists in close proximity to PCCs. Expressing complement proteins C3, C7, CFB, CFD, CFH, and CFI, these csCAFs are thought to regulate complement activity and contribute to inflammation, but notably, these cells are in their highest concentration in early-stage PDAC with declining levels throughout its progression (74). A smaller subset of CAFs, known as antigen-presenting CAFs (apCAFs), express MHC II genes and induce CD4 T-cell activation, characterized by CD25 and CD69 expression, setting them apart from traditional antigen-presenting cells they lack CD80/CD86 (75). These apCAFs are believed to play a tumorigenic role in the production of regulatory T-cells (68). One other niche subset of CAFs includes metabolic CAFs (meCAFs). Phenotypically distinct from other CAFs, meCAFs present as a poor prognostic factor with a paradoxical increased responsiveness to immunotherapy (76). PLA2G2A is associated with higher CD8+ T-cells silencing but looser “hot” TME, PLA2G2A may function as a potential immunotherapeutic target in this regard (77).

CAF functions extend to impacting tumor metastasis within the PDAC TME through cell-cell interactions. Heparin Sulfate Proteoglycan 2 (HSPG2) exhibits increased expression in the context of paracrine signaling between p53+ PCCs and CAFs (78). HSPG2, or perlecan, is a complex proteoglycan present in the ECM. It displays the ability to separate cell layers and, when broken down, can additionally engage in growth factor signaling(79, 80). CAFs’ role in remodeling the ECM and facilitating tumor cell migration is evidenced by the upregulation of focal adhesion kinase (FAK) in these cells. FAK’s elevated presence aids in the active reconstructing of the ECM and promotes the tumor cell migration of differentiated cancer phenotypes (81).

The role of CAFs extends to cancer stem cell (CSC) support and chemoresistance, with specific subsets contributing to these processes. Notably, CD10+GPR77+ and CD44+ CAFs, when co-cultured with cancer stem cells and PDAC cancer cells, exhibit increased tumorigenesis, chemoresistance, and disease recurrence (82, 83). Demonstrating the cooperative role of CAFs with PDAC cancer cells in tumor progression and chemotherapeutic resistance, the TME must be considered from the perspective of the malignant redefining the role of benign pathways.

Appropriate therapeutic approaches demand evaluation of the cause-effect role that upstream pathways like myCAF knockouts and SHH pathways have on PDAC outcomes. SHH inhibition, leading to increased ratio of iCAFs to myCAFs, led to shorter overall survival in patient treatment (84). This may indicate the undue reliance on SHH signaling by fibrotic CAF phenotypes with a resulting increase in iCAFs that promotes tumorigenesis. Inhibition of iCAF activation through targeted measures via CXCL12 and CXCR4 inhibition increases the immune response in PDAC (85, 86). Unspecified targeting of these activated support cells will lead to unforeseen consequences for the patient if additional consideration for the effect these individual subsets have on the impact of PDAC outcomes. The variability in function and origin within this cell type implicates the role of signaling molecules and crosstalk with PCCs as major players in activation. Next steps should continue investigating the balancing mechanism of these effector proteins to determine the optimal strategy for successful clinical applications.

## Stroma

The pancreatic tumor is primarily composed of a desmoplastic, scar-like capsule that is a result of extracellular matrix (ECM) production and turnover by both PCCs and resident stromal cells. Included in this environment is a variety of proteins secreted by supporting cells with malignant reprogramming. Although once an appropriate response to inflammatory signaling and tissue injury, TME remodeling becomes an imbalance of indefinite fibrosis, alterations in ECM protein, and immune suppression. Such remodeling involves changes in protein deposition, post-translational modifications of the protein deposited, and breakdown of tissues surrounding the TME (87). Components of the desmoplastic environment that play a role in the malignancy of PDAC include the various proteins, the hypoxic environment, and the high pressure that innately resists outside intervention.

The capability of cancer cells and the tumor microenvironment to expand into the surrounding tissue and begin its malignant expansion has largely pointed back to the function of angiogenesis (88). Previous literature established the role of angiogenesis in solid tumor models as bridging the gap between hyperplastic tissue and neoplastic tissue as an indicator of reprogramming non-malignant cell populations for cancer survival. Different models of angiogenesis vary between recruitment of endothelial cells for neovascularization, cellular reprogramming to utilize undifferentiated cancer cells to mimic vessel function, and malignant spread that preserves the non-malignant tissue known as vascular co-option (89). PDAC differentiates itself from other solid tumor malignancies due to its characteristic nature of overall *decreased* vascularity. Desmoplasia with the abundance of fibroblast/CAFs and the dense ECM produced by PSCs lead to compression of microvasculature in the microenvironment and overall hypovascularity of the TME. Pancreatic cancer cells and pancreatic stellate cells produce hallmark transcription factors known to be involved in angiogenesis: namely NFκB, Sp, STAT3, and HIF-1α (90). This increased angiogenic signaling stops short of complete vascular perfusion as prior studies investigating blood flow through pancreatic tumors have shown significantly less perfusion through the TME vs surrounding pancreatic tissue with overall increased metabolism (91). Recent literature has suggested microvilli branching off of microvessels may be a unique supply within the peripheral of the PDAC TME for metabolic fuel unlike less dense tumor microenvironments (92).

Systematic review of clinical trials investigating anti-VEGF treatments found no clinically meaningful benefit in targeting angiogenic factors that have seen previous success in other solid tumor malignancies (93). Alternatively, preclinical models investigating “normalization” or reestablishing vascular supply to the hypoxic PDAC TME have shown increased efficacy in traditional chemotherapeutic agents as a means of increasing delivery and decreasing the hypoxic environment, a known mechanism of chemoresistance and tumorigenesis (94). Given the physiologic nature of the PDAC’s vascular supply, further investigation that looks at disrupting the fibrotic stroma with concurrent immunologic therapies may prove to have greatest therapeutic benefit moving forward.

The ECM is not strictly tumor promoting as previous studies that indiscriminately target myofibroblast cells led to increased metastasis (95, 96). Therefore, a combination of proteoglycans, collagen fiber, and other support structures that make up the ECM play a dual role of inhibiting and promoting depending on the individual molecule. Further investigation into the ECM with proteomic data reveals that the PDAC TME comprises upwards of 300 proteins with a mixture of collagens, glycoproteins, proteoglycans, ECM regulators, ECM-affiliated, and secreted factors (97). Within this composition lies the variability between normal pancreatic tissue and PDAC in the relative expression of ECM proteins. From early-stage PanIN to later stages of PDAC, there is a relative shift in the exact proteins expressed within the ECM (97). Distinct proteins and their prognostic associations within the ECM are continually studied to understand the role of ECM signaling and physical mechanisms in the PDAC TME.

### Decorin

Decorin expression, a small leucine-rich proteoglycan (SLRP), is generally linked with good overall outcomes with its detection in the extracellular matrix. Expressed by PSCs and to some extent PCCs, Decorin is shown to be downregulated in relative concentration compared to other ECM proteins secondary to TGF-β1 signaling by PCCs (98). Decorin possesses the role of a tyrosine kinase inhibitor for various extracellular signals that modulate the overgrowth of surrounding tissue (99). Examples of such modulation include anti-angiogenic, anti-metastatic, pro-inflammatory, and anti-proliferative properties (100–103). As such, decorin has become a predictive marker of clinical outcomes as lower intra-tumoral concentrations are correlated with poorer patient survival (104). Although previous literature indicated that decorin expression may be elevated in PDAC, it was further shown the isoform with anti-tumor properties was not present in large quantities within the desmoplastic environment.

### Lumican

Lumican, an SLRP present in the ECM, has been associated with prolonged survival in post-surgical patients, primarily attributed to its poorly understood anti-tumorigenic effects (105). Similar to Decorin, Lumican, although produced in excess due to the increased ECM production, is decreased by TGFβ1 signaling by PCCs (98). Evidence suggests that it inhibits the signaling of EGFR through extracellular binding with resultant downregulation of EGFR/ERK pathway to reduce PDAC cell proliferation and increase apoptosis (106). Notably, Li et al. observed that when Lumican was completely knocked down in *in vivo* models, it led to enhanced proliferation (106). Interestingly, overexpression or exogenous administration of excess Lumican did not significantly affect proliferation (107). Furthermore, Lumican has been found to exhibit suppressive and promotional roles in various other tumor types, making it a subject of ongoing investigation to pinpoint its precise function in PDAC (99).

### Hyaluronan

In typical tissue environments, glycosaminoglycans (GAGs) constitute a large portion of the ECM. GAGs routinely bind large volumes of water molecules to structure the ECM and allow diffusion of materials. Hyaluronan, a common GAG molecule that composes a sizable portion of a stable ECM, is overexpressed in the TME to the point that the GAG to water ratio becomes oversaturated and the fluid-like environment becomes a higher pressure, gel-like environment (43). This increased hydrostatic pressure promotes a surrounding area of minimal poor vascular growth and present vascular collapse leading to additional hypoxia. *HAS1* and *HAS2*, the genes that produce Hyaluronan, are highly expressed in iCAF cells as determined through single-cell RNA sequencing (68). Interestingly, a primary driver of the NF-κB pathway, IRAK4, is shown to directly promote the expression of *HAS2* in both PCCs and CAFs exposed to PDAC conditioned media (108). On top of increased hyaluronan production, *HAS2* upregulation dramatically reduced CD8 T-cell presence within the PDAC TME, this helps illustrate the role immunosuppressive role of hyaluronan. Previous interventions analyzing direct targeting of Hyaluronan in the TME led to significant improvements in overall survival in mouse trials as it increased perfusion and decreased interstitial fluid pressure allowing penetration of gemcitabine (5). Although short-term destruction of hyaluronan leads to temporary therapeutic improvements in the fibrotic TME; without continual treatment, production from PCCs and CAFs result in resurgence of hyaluronan and interstitial fluid pressure to before-treatment values (109). Administrative strategies and therapeutic timing may need to be reconsidered as Phase III clinical trials as late as 2020 examining the use of PEGPH20, a long-acting hyaluronidase enzyme, in conjunction with Nab-Paclitaxel plus Gemcitabine found no significant increase in overall survival (110). Possible limitations within this trial and our understanding of targeting such proteins may be the metastatic course of the disease, as the greatest impact from such therapies may be seen earlier in the disease process.

### Matrix metalloproteases

Although previously addressed as playing a more significant role within the PDAC TME, Matrix Metalloproteases (MMPs) have seen less investigation and research in recent years. The function of the MMP family is overarchingly to act as a protease within the extracellular environment to degrade varying ECM materials with further subclassification based on the targeted proteins. The major subsets based on targeted substrate include collagenases, gelatinases, stromelysins, matrilysins, membrane-type MMPs, and other MMPs (111). Although the purpose is to break down protein, MMPs additionally function in tissue remodeling, cellular differentiation, and proliferation (112). Among the MMPs, 2, 7, and 14 have routine support within the literature as pro-tumorigenic effects on the PDAC TME with some additional debate of MMP-9. MMP-2 and MMP-9 are within the gelatinase category, with MMP-2 known to be activated by TNF-α and TNF-β with expression from endothelial cells. MMP-9, although elevated in PDAC progression and believed to activate TGF-β signaling, has historically been the target of anti-invasive therapies (113) Clinical trials that targeted MMP-9 noticed counterintuitive responses with poorer overall survival. A phase III clinical trial in 2003 comparing a broad MMP inhibitor, BAY 12-9566, saw a lower median overall survival compared to gemcitabine in 277 patients (114). Additional pre-clinical research investigating systemic knockout models of MMP-9 witnessed secondary effects of poorer immune response leading to increased overall tumor invasiveness. Consequently, selective inhibition within the PDAC TME may remain beneficial, but systemic administration of a non-targeted inhibitor has proven to be deleterious. Evolving therapeutic modalities discussed later may provide the opportunity for such selective delivery that allows targeting proteins with vital normal tissue functions similar to MMP-9.

MMP-14, also known as MT1-MMP, is a Membrane-type Metalloproteinase expressed on fibroblasts and cancer cells. A major effect established by MMPs within the TME is their use in tumor invasion through the breakdown of surrounding tissue, particularly the role of MMP-14 and its membrane expression directly linked to invadopodia function and metastasis (115). Additionally MMP-14 is shown to activate TGFβ-1 by means of direct activation and enzymatically releasing TGF-binding protein-1 in the ECM (116, 117). The direct impact of increased TGF-β being increased collage production and PCC metastatic potential within the PDAC TME (118, 119). Expression of intracellular Metalloproteinase CT Binding Protein-1 protein and extracellular Tissue Inhibitor of Metalloproteinase-2 have demonstrated a reduction in invadopodia formation (115, 120). These proteins offer potential therapeutic targets in delivery mechanisms that allow local expression and inhibition rather than the general administration of exogenous proteins that lack specificity. Previous clinical therapies investigating inhibition of MMPs resulted in no significant benefit; similarly, due to difficulties in targeting particular MMPs and the potential need for early PDAC stage application as a potential time of efficacy for such therapies.

### Other ECM proteins

Additional ECM proteins with an established role within the PDAC TME but still require further investigation include the following discussed. Asporin, another SLRP present within the ECM, is released by both PSCs and pancreatic cancer cells. It is known to activate signaling through the CD44/NFκB pathway, promoting EMT and metastasis in pancreatic cancer (121). An additional SLRP of interest, Keratocan, is overexpressed in the PDAC TME and has been linked to a poorer prognosis and concurrent downregulation of p53 (107). The presence of Meflin, produced by cancer-associated fibroblasts, has been associated with additional regulation of PDAC tumorigenesis, yet further exploration is needed to fully understand its impact and potential use in therapeutic intervention. (62). These ECM proteins, although possessing established roles, require deeper investigation to understand their implications with the landscape of PDAC progression.

## Myeloid-derived suppressor cells

Further consideration of the role inflammation plays in PDAC oncogenesis points to the existence of MDSCs as a major player within the TME. Lacking in *in vivo* data for the initial transition of pancreatic tissue to PDAC, it is understood that an early, increased presence of MDSCs is indicative of oncogenesis (122). In the event of an injury to the pancreas, signaling molecules such as CSF-1 and CSF-2 will induce differentiation of hematopoietic stem cells into immature myeloid cells (IMC) and migrate to the site of injury. This recruitment’s initial intention is to provide precursor innate immune cells for additional support in the inflammatory process. The chronic inflammatory environment that functions as a precursor to the PDAC TME, serves to upregulate the generation and migration of MDSCs to the tumor environment as part of the inherent purpose of MDSCs to suppress inflammation. Such environmental signaling includes IL-6/IL-4, CSF-2, COX2, S100A8/9, IL-1β, and IFN-γ. A combination of both IL-6 and CSF-2 not only recruit MDSCs but induce miRNA generation to prevent them from further differentiation (123). Early evidence of primary tumor removal resulted in decreased circulating levels of IMCs as our early understanding investigated the role of recruitment of these anti-inflammatory immune cells (124). Although promising, transcriptomic analyses of lung cancer populations have exposed the limitations in evaluating blood samples as correlates of TME cell populations as many of the TME-specific populations are potentially differentiated once reaching the site of malignancy (125). Although they display numerous characteristics similar to other innate immune cells, MDSCs in humans are identified by surface receptors including CD33, CD11, and Ly6c (126). Two major MDSCs have been noted in the pancreatic TME, including the granulocytic MDSCs (PMN-MDSC) or Monocytic MDSCs (M-MDSC). Besides surface markers, M-MDSCs will feature CD14/15 where otherwise absent on PMN-MDSCs, and path of origin being their differences, the primary role each plays in the TME is based on their specific secretory molecules. M-MDSCs will primarily exhibit increased NO production while PMN-MDSCs will produce ROS, both of which reduce T-cell activity (127). Although there has been additional investigation into specific functions of each cell type, the overarching role of MDSCs remains the focus of this review.

Within the PDAC TME, exosomes released by malignant cells and malignantly programmed cells induce and upregulate transcription factors within MDSCs that further regulate and suppress the immune response(128). Both NF-kB and STAT3 have shown levels of upregulation within this context(129). Due to these mechanisms, MDSCs are shown to express elevated levels of arginase 1(ARG-1), an enzyme directly targeting L-arginine, and IDO-1/TDO, an enzyme targeting L-tryptophan to produce kynurenine. These two enzymes in combination work towards depletion of amino acids key in proliferation which indirectly suppress T-cell proliferation and potentially tumor proliferation (130). Additional effects of ROS and RNS production has shown to include decreased reactivity of MHC 1 molecules binding ability with cytotoxic T-cells in tumor cell populations (131). Additionally, MDSCs are known to release IL-10 and TGF-β for regulatory T-cell activation via CD40 (132). Receptor for advanced glycation end products (RAGE), a MHC class III receptor involved in recruitment and pro-longed survival of MDSCs, is shown to have increased expression PCCs and TME surrounding MDSCs. (133, 134). This receptor which responds to inflammatory signaling aims to sustain and recruit the MDSC population, prolonging their immunosuppressive effects on the TME. This along with continual recruitment of new MDSCs by the CXCR4/CXCL12 signaling pathway promotes indefinite immunosuppressive regulation by the PDAC TME (126). Decreased recruitment of MDSCs to the PDAC TME is showing a promising angle in novel therapeutic strategies by inhibiting the signaling pathways key in MDSC recruitment. As it stands, MDSCs serve as a major barrier in immunotherapies designed to utilize the body’s own immune system as they provide significant local-regional immune suppression.

## Tumor-associated macrophages

Macrophages are demonstrated to be the frontline response within the immune system to instances of infection and injury (135). Monocytes make their way to the site of inflammation via VCAM-1/VLA-4 binding and CCR2/CCL2 migration (136). In addition to migratory monocytes, resident tissue macrophages simultaneously respond to initial insults. Alluded to earlier within this review, we discussed the balance and resolution of inflammation and how loss of such balance leads to a lack of resolution and the formation of chronic inflammation. M1 and M2 macrophages strike a homeostatic balance between pro-inflammatory and anti-inflammatory mechanisms based on cellular signaling and cytokine production. Within the early stages of an inflammatory process, M1 macrophages, induced by inflammatory markers such as IFN-γ and CSF-2, will release IL-1β, IL-12, IL-6, IFNα/β, and TNF-α in order to magnify the immune response (137). Over time as the initial infection subsides and repair mechanisms are prioritized, M2 cells will begin to dominate with the resolution of the infection while expressing immune suppressing and remodeling factors such as VEGF, IL-4, TGF-β, and IL-10 (138).

In the context of the PDAC TME, monocytes that are brought to the site maintain a similar initial response of differentiation and immunologic reaction to the pathology, but TME signaling provides an environment susceptible to imbalance. Cytokines and chemokines released by the TME such as CXCL8 binding CXCR1/2 on monocytes result in consistent recruitment for additional macrophages (139). A key chemokine mentioned previously, CCL2, is upregulated in the context of hypoxic environments secondary to HIF-1α promotion which is continuously produced within TME to recruit macrophages (140). These nonspecific monocytes and macrophages become polarized to favor the role of immunosuppressive M2s due to PCC signaling molecules such as IL-13 and TGF-β with most of these simultaneously suppressing M1 macrophage activation due to the nature of the macrophage’s polarity (141, 142). M2 macrophages are also shown to aid in metastatic properties of PDAC as previous literature indicates the role of granulin release by metastatic macrophages as associated with hepatic stellate cells fibroblastic activation and satellite TME formation (143). CSF-1 secretion by the TME directly affects repolarizing TAM M1s into M2s as inhibition of the receptor pathway in cancer cell models reduced the M2 to M1 decreased (144). The tumor microenvironment while prioritizing the formation of immunosuppressive M2s will also actively suppress the role M1s have in pro-inflammatory signaling. The general presence of TAMs within the TME points to the role of malignant transformation and rewiring of the inflammatory process in PDAC. Although CD163+/206+ are markers specific to M2 macrophages and is indicated in worse overall survival; CD68+, a non-specific marker of macrophage presence, is also associated with poor overall survival, speaking to the heterogeneity of the macrophage population (145). Proper targeting of macrophages cells within the TME has previously shown a promising role in potential therapeutics with clinical trials and research focused on this process ongoing.

## Tumor-associated neutrophils

In the complex makeup of the TME, neutrophils assume a pivotal role, with their recruitment being partially mediated by CXCR1 and CXCR2 receptors on neutrophils (146). These receptors are activated by multiple signaling molecules including CXCL-1, CXCL-2, CXCL-5, CXCL-8, and IL-8 released by various constituents of this environment, including cancer cells, immune cells, and cancer-associated fibroblasts. Supporting this recruitment, CSF-3 and IL-17 are involved in facilitating the migration and proliferation of neutrophils, contributing to their presence at the tumor site (147, 148). Upon reaching the TME, neutrophils undergo distinct activation pathways. IFN-β signaling prompts the activation of N1 neutrophils, specialized cells that release critical CD8-T-cell activating signals such as TNF-α, CCL-3, CXCL-9, and CXCL-10 (149, 150). These signals help bolster the immune response and enhance the cytotoxic activity of CD8-T-cells.

Conversely, IL-35 and TGF-β, primarily released by regulatory T-cells (Tregs), trigger the activation of N2 neutrophils. Unlike N1 neutrophils, N2 neutrophils possess immune-suppressive characteristics. They release molecules such as CCL2/3/4/17, Neutrophil Elastase, MMP9, and elevated arginase, contributing to immunosuppression and TME remodeling that fosters tumor progression. These pro-tumor neutrophils exhibit altered gene expression, upregulating genes like *VEGFA, PLAU, LGALS3, PDE4D*, and *LDHA* (151). *PLAU*, for example, is significant for encoding urokinase plasminogen activator, an upstream activator of the c-MET pathway in cancer cells (152). The TME’s unique conditions, including tissue hypoxia and ER stress, can also influence neutrophils to differentiate towards pro-tumor subtypes. In addition, the PDAC TME provides signaling, including IL-17, that activates a cellular function called neutrophil extracellular trap (NET) in which granular webs are released into the ECM (153). NETs work in a host’s defense system to trap pathologic organisms as part of the innate immune system, but research shows NETs are triggered within the PDAC TME and implicated in tumor metastasis and functioning as a physical barrier in anti-tumor immune responses (154, 155). Although the contributions of neutrophils to the PDAC TME and their role in immunosuppression has remained been debated in previous literature, a clearer understanding of the mechanisms and signaling involved has challenged this notion. Like other support cells in the PDAC TME, recruitment and unbalanced differentiation into tumor-promoting phenotypes may be the mechanism with greatest therapeutic potential.

**Figure 1:**
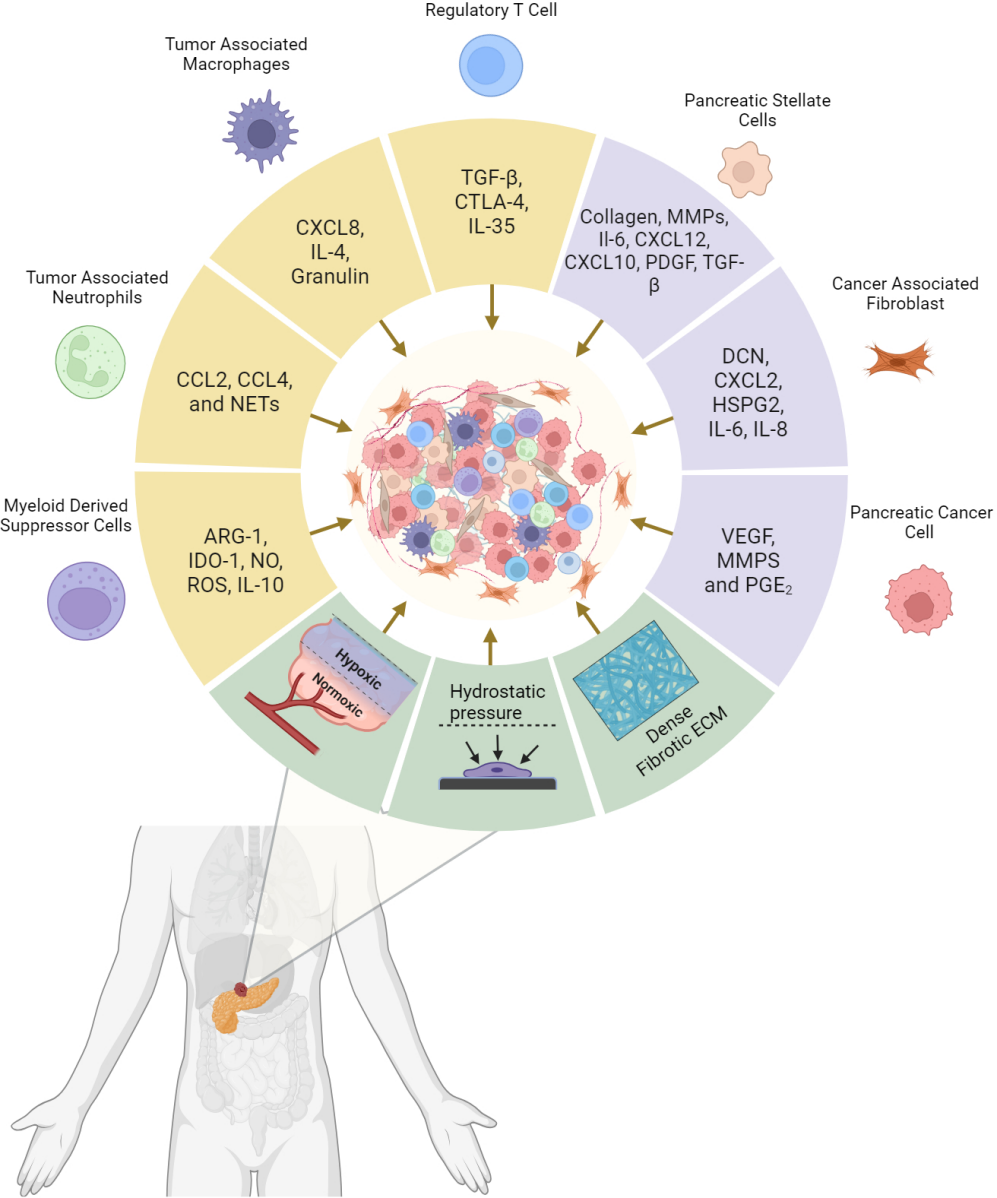
Pancreatic Ductal adenocarcinoma tumor microenvironment (TME) composition and the driving factors of tumor desmpolasia. Cytokines and tumorigenic signaling molecules are released by cancer cells, support cells, and immune cells that subsequently promotes the desmoplastic structure. In addition, mechanical elements stimulate these cells to perpetuate the environment and enhance further tumor growth. The resulting physical barrier and immune counterbalance are the critical factors responsible for systemic and immunotherapeutic resistance in PDAC. Created with BioRender.com.

## Regulatory T-cells

Regulatory T-cells function as a safeguard in normal inflammation. Regulatory T-cells act as a part of the natural immunosuppression meant to minimize the risk of autoimmune diseases and other overactive immune responses (156). The immunoregulatory role of Tregs in the TME limits the efficacy of the immune system in attacking patient’s cancer cells. Regulatory T-cells are defined by their presence of the transcription factor FoxP3, IL-2Receptor (CD25), and CTLA-4 with direct roles in down-regulation of CD80/86 antigen-presenting cells and reduction of the immune response (157).

Tregs are a factory of general immunosuppression through a variety of mechanisms. Consistently present even within early stage PanIN, regulatory T-cells are examined within proximity of the tumor cell environment with the additional presence of M2 macrophages (158). In addition to CTLA-4, regulatory T-cells comprise part of the cellular populations that release TGF-β for later stage immunosuppression. In addition to TGF-β, regulatory T-cells demonstrate the release of IL-35 and IL-10 as mechanisms of potent immunosuppression (159). Combined inhibition of both TGF-β and PD-1 shows a direct increase in cytotoxic T-cells, emphasizing the effects Tregs and other secretory cells have in the TME (160). Although a simple expectation of removing Tregs from the TME seems like first response to this, evidence of early knockout of Treg cells related to tumor progression rather than reduction. This in part was seen due to compensatory accumulation of other CD4+ immune suppressing T-cells and increased fibroblast-linked chemokines shown to recruit myeloid derived suppressing cells (158). Rather, mitigation and inhibition of Treg’s role in recruitment and migration of MDSCs through CCR1 inhibition showed promise in reducing carcinogenesis. In support of this, MDSCs are shown directly communicating with Tregs through physical contact to promote tumor progression potentially through the B7-H1 pathway, but recent evidence suggests that MDSCs and Tregs work synergistically through bidirectional recruitment and tumor-specific signaling (161, 162). In therapeutic applications, one of the largest insights gained in recent years refers back to the effect of low-dose cyclophosphamide on depletion of Tregs in the context of the TME, yielding stronger response to immunotherapy. Similar measures focusing on the targeting of upstream pathways that result in Treg recruitment or inactivation will serve as a basis for future points of investigation.

## Desmoplastic evolution

The culmination of malignant reprogramming, cellular interactions, and structural malconformations produces the fibrous, dense tissue in pancreatic adenocarcinoma. The physiologic insults and aberrant signaling of pancreatic cancer cells, combined with the unsuccessful resolution of inflammation, is the initiating factor for fibroblastic activation and programming. PSC and CAF deposition of collagen, hyaluronan, and other ECM proteins have a combined utility in most physical characteristics of desmoplasia (**Figure 1**). The dense collagen matrices and increased interstitial pressures present a physical barrier for bioavailability of cytotoxic immune responses and therapeutic agents. For such metabolic demands with minimal perfusion, hypoxic environments promoting ROS formation, cellular autophagy, and transcriptional upregulation of HIF consequently lead to additional therapeutic resistance. Although primarily functioning in the ECM, previously mentioned proteins such as Asporin, Keratocan, and Periostin show direct influence on PDAC cancer cell tumorigenesis by demonstrating roles in membrane receptor binding and transcriptional regulation. The combination of high pressure, poor perfusion, and dense fibrosis presents a desmoplastic environment that encourages pancreatic cancer cells to search for greener pastures. Survival pressures and interactions from the extracellular matrix combined with paracrine signaling from support cells amplify the drive for cancer associated EMT. This combined with the supportive role of signaling growth factors from activated fibroblasts, compounds the effects on tumor growth. Inflammatory markers enhance these effects while MDSCs, Tregs, and M2 macrophages simultaneously suppress Effector T-cells, Dendritic Cells, and CD8 Cytotoxic T-cells from targeting and killing tumor cells. Strategies for targeting the desmoplastic stroma have shown promise, with a greater focus on reducing pressure, increase vascularization, and increase the diffusive capacity of the ECM.

## Novel therapeutic strategies

### CAR-T-cell therapy

Among emerging therapies, Chimeric Antigen Receptor T-cell (CAR-T) therapy has long received a level of interest since its inception in 1993 (163). The technology utilizes the patient’s immune system by introducing synthetic antigen receptors capitalizing on antibody binding sites with the T-cell intracellular signaling pathways. The benefit of CAR-T therapies is that they are not subject to immune-evasive effects that cancer cells routinely exhibit. The foundation for effective CAR-T-cell therapies, exemplified by the anti-CD19 drug for B-cell ALL, Tisagenlecleucel, is targeting unique receptor proteins predominantly expressed in cancer cells (164). A major barrier to CAR-T-cell therapies in PDAC patients includes the lack of uniquely defining receptor proteins that would consistently represent PDAC. Current PDAC CAR-T therapies have focused on CEA, EpCAM, Mesothelin, and CD133, but PDAC’s immune exhaustion mechanisms and the fibroblastic barrier to tumor cell exposure have proven to be limitations in CAR-T application (165). A Phase I clinical trial investigating the use of CAR-T cells programmed to bind Mesothelin in chemotherapy-refractory PDAC patients noted such results with limited detection of CAR-T transcripts within the TME following administration, although minimal toxicity was noted (166). Additional approaches to CAR-T cell administration have shown benefit through loco-regional administration of therapy rather than intravenous with marked increase in therapeutic response in preclinical models with further exploration following in phase I clinical trials. (167)(NCT01373047). In addition to challenging targets, adverse events relating to the induction of some novel immune therapies include cytokine release syndrome, seen with rapid activation of cytokine signaling cascades. Generational changes in CAR-T technology have focused on increasing cytokine response and support signaling to maximize the response in recognizing cancer epitopes and activating the immune response while minimizing these adverse events (168). Current clinical trials are beginning to utilize these newer generations of CAR-T-cell therapy and hope to address such limitations while investigating combined therapeutic strategies that utilize CAR-T-cell therapy with complementary immunotherapies.

### Bispecific T-cell engaging antibody

Bispecific T-cell engaging (BiTE) antibody therapy, serves the purpose of activating the host immune response against specific cellular receptors that are either unique to or over-expressed on cancer cells. An example of an over-expressed receptor is EPCAM, an intercellular signaling receptor commonly seen on epithelial cells for cell-cell adhesion and communication. This over-expression is particularly notable in pancreatic cancer (169). MT-110 and Catumaxomab are two BiTEs designed for EPCAM, CD3 T-cell, and immune system activation against openly available EPCAM molecules to bind to (170). A phase 1 trial of MT-110 or soliton showed an unfortunately high rate (95%) of transfusion-related adverse events that warranted discontinuation of the investigative medicine (171). Besides off-target effects limiting applicability, most BiTE therapies target cell-surface proteins with limited efficacy for intracellular markers (172). Even while selecting patients with elevated levels of the designed target marker, IGF-1R, those in the phase II clinical trial for Istiratumab were not found to benefit meaningfully from the therapy in combination with standard of care (173). New research investigating HSV oncolytic viruses designed with Claudin 18.2 BITE targeting antibodies allow production of tumor-specific antibodies within the historically immunologically cold tumor microenvironment (174). Potential avenues worth exploring may be the combination of these novel immunologic therapies to maximize efficacy while minimizing off-target effects.

### Cancer vaccine therapy

Vaccine therapy focuses on stimulating the host’s immune system to antigens uniquely expressed or upregulated within the PDAC TME. Tumor vaccine therapies involves a variety of vehicles, antigens, and genetic information to relay novel epitopes to the adaptive immune system. Vaccine methods include cell-, peptide-, protein-, DNA-, exosome-, and micro-organism-based. Such therapeutic methods although promising within early preclinical research and clinical trials, proved largely unresponsive in most phase II trials to-date (175). The largest phase III pancreatic cancer vaccine trial to date investigated the use of telomerase peptide vaccine in 1062 patients with no significant benefit in overall survival compared to standard of care alone (176). Evolving as a modern therapy in part due to the implementation of the COVID-19 vaccine, mRNA vaccines provide the schematics for protein expression with local reconstruction presenting the desired end product (177). Cancer mRNA vaccine therapy derives from using a patient’s tumor cell’s transcriptome, the mRNA present within the pancreatic cancer cells, to develop novel targets unique to the patient’s cancer. The benefits of mRNA vaccines over previous vaccines goes back to the large-scale utilization and manufacturing experienced during the COVID-19 pandemic. The limitations of modern research include our limited understanding of all potential targets within the PDAC TME. Some limitations in this therapy type go back to the immunosuppressive environment with a lack of immune system invasion through the PDAC TME. A recent trial utilizing personalized mRNA Neoantigen vaccines in addition to anti-PD-L1 (atezolizumab) and conventional chemotherapy in the adjuvant setting induced substantial T cell activity and demonstrated an increased recurrence-free survival in the phase I feasibility trial; although this patient population was limited to resected tumors and up to 20 neoepitopes within each vaccine (178). Based on these successful results, a phase 2 study is currently recruiting patients.

Bypassing the local immune suppression within pancreatic cancer remains a hurdle of this therapy as activated cytotoxic T-cells still must confront the TME, but methods of enhancing such therapies are a simultaneously evolving field. Codelivery with cytokine mRNA, such as IL-12, IL-15, IFN-α, or other immunostimulatory molecules, directly to tumor sites or in circulation with neoantigen mRNA may prove to be an alternative method to overcome these immunosuppressive barriers (179, 180).

### Antibody-bound drug conjugates

Another emerging therapeutic approach involves the use of antibodies targeting of cancer-specific membrane molecules combined with a linker molecule and cytotoxic agent (181). These antibody-drug conjugates (ADCs) have been actively developed over the last two decades with FDA approval for Brentuximab Vedotin and Ado-trastuzumab emtansine for use in Hodgkin Lymphoma and breast cancer respectively (182, 183). Similar drugs are under investigation in PDAC with developmental ADCs targeting Anti-glypican-1, HER3, Claudin18.2, and CEACAM6 (184–187). A Phase II clinical trial investigating the use of Anetumab-Ravtansine, an ADC targeting mesothelin expressing pancreatic tumors, in 14 patients evaluated for toxicity and progression free survival. Progression free survival was a median of 63 days and approximately 11 of experienced serious adverse events (188). Some limitations in the past with this therapeutic mechanism included late onset-drug toxicity and “bystander killing” by the effects of the cytotoxic chemical component acting on surrounding normal cells. Although a benefit of this unintended effect may be the untargeted destruction of tumor-associated support cells and tumor-suppressive immune cells that populate the environment. In addition, due to the dense, desmoplastic stroma of the PDAC TME, limitations in efficacy may derive from regulations in antibody drugs properly reaching most cancer cells (189). New clinical trials are investigating a combination of treatments along with ADCs to maximize their therapeutic efficacy. Anetumab-Ravtansine combined with Gemcitabine and Nivolumab was showing signs of stable disease in a progressing Phase Ib trial with a now expanded investigation into the responding treatment regimen (190). Improvements have helped to reduce these effects with continued efforts to implement a potentially effective drug delivery mechanism that has shown promise with other solid tumors. Given the potential for potent targeted cytotoxicity, this avenue of research may benefit greatly from stromal modifying agents allowing for improved delivery to cancer cells.

### Neoepitope TIL therapy

Additional therapeutic trials are investigating the use of patient-derived tumor invading lymphocytes (TIL) in combination with autologous pancreatic cancer cell exposure for neoepitope generation (191). A current phase II trial is looking at approximately 330 metastatic cancer patients is investigating the use of TILs with concurrent use of immunotherapy targeting anti-PD1 to maximize the immune response (NCT01174121). Previous cases of TIL therapy showcase targeting of tumor-specific mutations that are unique to the patient’s PDAC cells. One example includes TIL directed towards *KRAS* G12D oncogene with conditioning immune modulating infusions tocilizumab and cyclophosphamide before administration of TIL therapy. The patient demonstrated metastatic regression and physiologic response to the therapy but ultimately succumbed to the disease (192). Engineering one’s immune system as the therapeutic model provides excellent potential for personalized medicine with targeting potentials, but immune evasion and inherent “cold” environment of PDAC requires additional means to potentiate this therapy.

### Oncolytic virus and other microbial therapies

Given PDAC’s immunosuppressive environment as discussed previously, novel therapies that target recruitment and activation of the immune system and breakdown of the physical barriers may play a key role in future treatments. Lytic viruses designed specifically to target cancer cells have become a recent field of interest for oncologic research. T-VEC, a strain of HSV designed to target advanced melanoma, was approved by the FDA in 2015 (193). Alongside tumor-specific targeting with genetic engineering, oncolytic viruses wield the potential to serve as a carrier of genetically significant proteins that may assist in combating the TME (194). An adenovirus loaded with Relaxin, CSF-2, and IL-12 variants demonstrated a reduced PDAC TME with a concurrent increase in immunoresponsiveness (195). Relaxin (RLX), an exogenous peptide hormone, has previously shown evidence of expression during the physiological reduction of fibrotic structures (196). Targeting the tumor microenvironment (TME) directly with relaxin nanoparticles has demonstrated the improved therapeutic efficacy of Gemcitabine within *in vivo* models (197). Research trials utilizing VCN-01, an adenovirus expressing PH20, and Pelareorep, a reovirus, in disrupting the TME and awakening the immune response are underway as this novel therapy continues to evolve (198, 199). Moving forward, dual targeting of the PDAC TME and pancreatic cancer cells may prove efficacious in maximizing the utility of this novel therapy and overcoming therapeutic challenges of PDAC. Our group and others are investigating this strategy to optimize PDAC response to immune oncology approaches and other systemic treatments. In addition to viruses, efforts are underway to leverage the tumor-targeting capability of some bacteria to convert PDAC into an immunologically hot milieu. Notably, a genetically attenuated strain of salmonella typhimurium expressing IL-2, Saltikva, recently received FDA fast-track designation for the treatment of metastatic pancreatic cancers following phase 2 clinical studies (NCT01486329), highlighting new opportunities ahead for bacteria-based cancer therapeutics.

## Future directions

PDAC remains a lethal disease due to many factors that prevent adequate delivery to, and targeting of, pancreatic cancer cells. Current literature highlights the evident role the TME has in the initiation, progression, and treatment intractability of PDAC. The cellular components of the TME, combined with the extracellular environment and signaling pathways with surrounding tissues, shed light on the intricate complexity of how PDAC develops and the complex nature of resistance to innate and external anti-tumorigenic mechanisms. As the field evolves to better understand the dynamic interactions within the TME, novel therapeutic strategies of drug delivery and unique targets will unravel this disease. Nevertheless, critical gaps in our knowledge demand further research to reveal the precise mechanisms underlying TME-tumor interactions, taking into consideration the heterogeneity that characterizes both the tumor and the TME. Integrating -omics data, advanced imaging techniques, and sophisticated *in vitro* and *in vivo* models will advance our understanding of these intricate relationships. Novel therapeutic modalities will be refined based on more comprehensive understanding of mechanisms involved with primary and secondary resistance to systemic and immuno-oncology approaches. Moreover, future directions will be more focused on multimodality precision approaches, targeting different aspects of tumors, including cancer cells, microenvironment, and immune system.

## Author contributions

CH: Writing – original draft. BN: Writing – review & editing. CC: Writing – review & editing. MT: Writing – review & editing. JC-B: Writing – review & editing. JW: Writing – review & editing. OM: Conceptualization, Supervision, Writing – review & editing.
